# BOND: Basic OligoNucleotide Design

**DOI:** 10.1186/1471-2105-14-69

**Published:** 2013-02-27

**Authors:** Lucian Ilie, Hamid Mohamadi, Geoffrey Brian Golding, William F Smyth

**Affiliations:** 1 Department of Computer Science, University of Western Ontario, London, ON, N6A5B7, Canada; 2 Department of Computing and Software, McMaster University, Hamilton, ON, L8S 4K1, Canada; 3 Department of Biology, McMaster University, Hamilton, ON, L8S 4K1, Canada

**Keywords:** DNA oligonucleotide design, Microarray, Spaced seeds

## Abstract

**Background:**

DNA microarrays have become ubiquitous in biological and medical research. The most difficult problem that needs to be solved is the design of DNA oligonucleotides that (i) are highly specific, that is, bind only to the intended target, (ii) cover the highest possible number of genes, that is, all genes that allow such unique regions, and (iii) are computed fast. None of the existing programs meet all these criteria.

**Results:**

We introduce a new approach with our software program BOND (Basic OligoNucleotide Design). According to Kane’s criteria for oligo design, BOND computes highly specific DNA oligonucleotides, for all the genes that admit unique probes, while running orders of magnitude faster than the existing programs. The same approach enables us to introduce also an evaluation procedure that correctly measures the quality of the oligonucleotides. Extensive comparison is performed to prove our claims. BOND is flexible, easy to use, requires no additional software, and is freely available for non-commercial use from http://www.csd.uwo.ca/∼ilie/BOND/.

**Conclusions:**

We provide an improved solution to the important problem of oligonucleotide design, including a thorough evaluation of oligo design programs. We hope BOND will become a useful tool for researchers in biological and medical sciences by making the microarray procedures faster and more accurate.

## Background

DNA microarrays are a very useful tool that has become ubiquitous in biological and medical research. Their ability to simultaneously measure expression levels of thousands of genes depends essentially on the availability of DNA oligonucleotides, or probes, that bind specifically to their targets. A DNA oligonucleotide, henceforth *oligo*, is a single-stranded piece of DNA that uniquely binds to a given region, called *target *(e.g., a gene). An oligo can be *short* (20-25bp) or *long *(50-70bp); we focus in this work on long oligos as they provide increased performance [1].

The problem of designing high quality oligos has received considerable attention by the research community and quite a few software programs have been developed for designing oligos [2-21]. The reader is referred to the survey of [1] for a detailed description of the best existing such programs.

The oligos designed by these programs have to meet several conditions. The first is *sensitivity*: oligos have to bind strongly to their targets at similar temperature. Sensitivity is achieved by ensuring that their melting temperatures [22] are not far from each other, with implications on the GC content, and by avoiding secondary structures that would make the oligos bind onto themselves instead of targets.

The second, and much more difficult to fulfill, condition is *specificity*, that is, the oligos must not bind to non-targets. Avoiding cross-hybridization is the most important, and the most difficult, issue for oligo selection. Precise criteria have been established, and widely adopted by the community, to ensure non-cross-hybridization [23,24]; they are often referred to in the literature as *Kane’s conditions*: 

(*C*_75_) the overall complementarity with non-targets should be less than 75% and

(*C*_15_) there should be no contiguous complementary region with non-targets of length 15 or more.

Technically, condition *C*_75_ means that the Hamming distance between the oligo and any non-target should be less than 75% of their length. Oligos satisfying these conditions will be called simply “good”, whereas those that do not will be called “bad.” The search for specific oligos implies detection and elimination of regions that are similar. Therefore, many programs employ BLAST [25], the most widely used tool for similarity search. We show that the specificity that can be achieved this way is quite low. While condition (*C*_15_) can be easily fulfilled using text indexes, as done by PICKY [11], condition (*C*_75_) is much more difficult. The only program that achieves good specificity is YODA [17]. However, it employs an exhaustive search that is too slow to be used for most datasets.

Other conditions the oligo design programs have to meet are *coverage*, that is, designing oligos for as many genes as possible (note here that some genes cannot have their own oligos, due to high similarity with others) and *speed*; they should not only run in reasonable time but also be able to deal with datasets of any size. It turns out that none of the existing programs meet all these criteria.

We introduce a new approach with our software program BOND (Basic OligoNucleotide Design), that enables us to compute highly specific DNA oligos, for all the genes that admit unique probes, while running orders of magnitude faster than the competing programs. BOND is implemented in C/C++ and OpenMP, does not use any external software, is easy to use, and it allows the user to adjust a variety of parameters.

The same approach enables us to introduce as well an evaluation procedure that correctly measures the quality of the oligos. Extensive comparison is performed to prove our claims.

## Methods

### Oligo design, similarity search, and BLAST

An oligo binds to a non-target when they share considerable complementarity, as described by the conditions (*C*_15_) and (*C*_75_). That means, the reverse complement of the oligo and the non-target have substantial similarity and hence the search for specific oligos implies detection and elimination of regions that are similar. Therefore, many programs employ BLAST [25,26], the most widely used tool for similarity search.

The BLAST program has been enormously successful for similarity search (the two papers have received together over 88,500 citations to date) however, it is ill suited for oligo design since in this case exhaustive search is needed and BLAST misses many similarities. Doubts about using BLAST for this purpose have been expressed before [11,17] but the impact on the quality of the oligos produced has never been properly quantified. We provide such an evaluation in this section.

**Figure 1:**
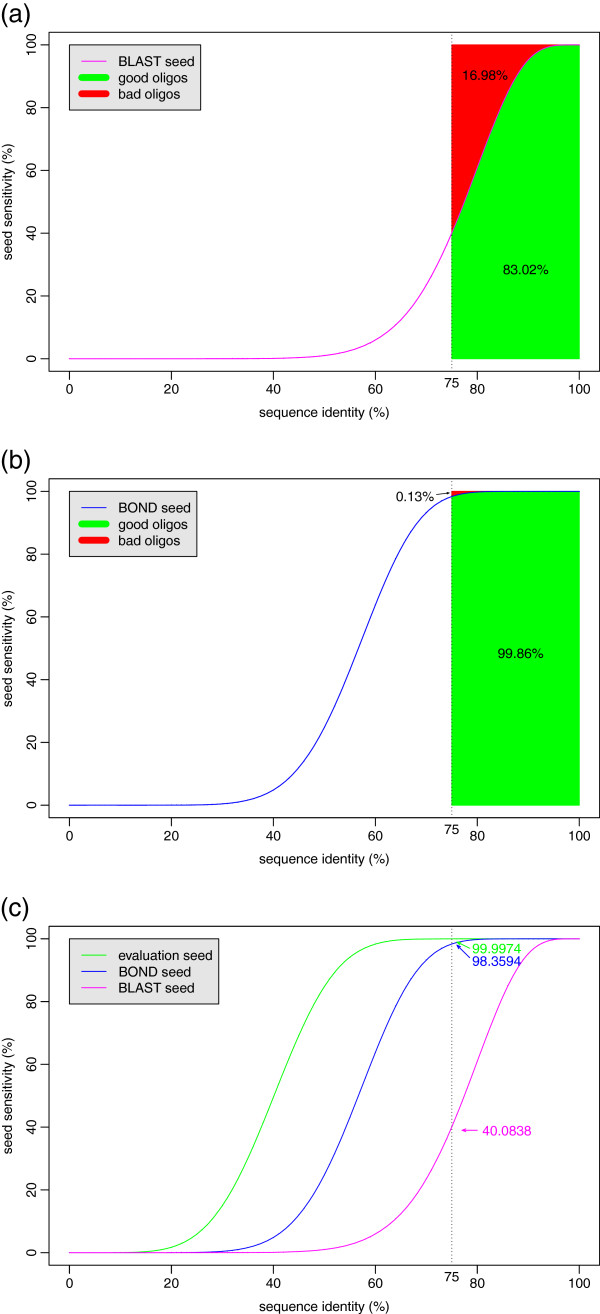
**Seed sensitivities. **The sensitivity of three seeds: **(a) **BLAST’s seed (used by most software programs), **(b) **BOND’s seed, and **(c)** evaluation seed (used in our evaluation procedure) are plotted against sequence identity. The red area above the curves in (a) and (b) shows the oligos that have identity 75% or more with non-targets but are not identified by the seed, whereas those inside the green area are identified by the seed. BLAST’s seed fails to identify 16.98% of bad oligos whereas BOND’s seed only 0.13%. The sensitivity of all three seeds are shown in the (c) plot, where their sensitivities at 75% sequence identity are shown; BLAST’s is only 40%. In all plots the length of oligos is assumed to be 50.

BLAST works by the “hit-and-extend” principle where a hit consisting of 11 consecutive matches is extended attempting to identify a local similarity. The 11 consecutive matches form what is called a *seed*. We plot in Figure 1(a) the probability (called “seed sensitivity”, see next section for a precise definition) of BLAST’s seed to identify the similarity of oligos with non-targets as a function of their sequence identity. According to condition (*C*_75_), we are concerned with the shaded part of the graph, where sequence identity is 75% or larger. The red part represents missed similarities and the green part those that are found by BLAST. Approximately 17% will be missed, which explains clearly why BLAST is not suitable for oligo design. In addition, in order to improve the speed, not all hits are investigated, thus bringing the probability of detection even lower and failing to fulfill condition (*C*_15_) as well.

The above reasoning applies in principle to any hit-and-extend method that uses consecutive matches as hits, assuming the hits are at least as long as the BLAST seed; 16 out of 20 programs listed in [1] use some form of consecutive matching.

### Spaced seeds

It has been noticed already in [27-29] that spaced matches provide better sensitivity but it was in [30-32] that research on finding the best spaced seeds with such properties was started. The first similarity search software program to use spaced seeds was PatternHunter [31]; it used the seed 111⋆1⋆⋆1⋆1⋆⋆11⋆111. For comparison, the contiguous seed of BLAST is 11111111111.

A spaced seed *s *is a sequence of 1’s (matches) and ⋆’s (don’t care positions). The number of 1’s is the *weight *of a seed and the total number of symbols is its *length*. Assuming a Bernoulli model [33], an alignment can be modelled as a sequence *R* of 1’s, for matches, and 0’s, for mismatches. A *hit *of *s *is a region of *R *of the same length as *s *such that 1’s in *s *correspond to 1’s in *R*. Given the length *N *of the alignment and the sequence identity *p *(percentage of expected 1’s in *R*), the *sensitivity* of *s *is the probability that *s *hits *R*.

A multiple spaced seed [33] consists of several spaced seeds. Its sensitivity is computed similarly. We say that a multiple spaced seed consisting of *k *seeds of weight *g *is a w*g*s*k *seed. For instance, PatternHunter has a w11s1 seed (one seed of weight 11) whereas PatternHunterII has a w11s16 seed (16 seeds of weight 11). Multiple spaced seeds can increase dramatically the sensitivity. As an example, the sensitivity of the BLAST’s, w11s1, and w11s16 seeds mentioned above, for *N *= 64 and *p *= 70 *%*, is 30%, 47%, and 92%, respectively.

Increasing the weight decreases the sensitivity but increases the specificity. To have both sensitivity and specificity high, we need to increase the number of seeds. The crucial observation [33] is that doubling the number of seeds has an effect on sensitivity that is similar to reducing the weight by one. Since the running time is proportional with the number of seeds and the number of random hits decreases four times when increasing the weight by one, both sensitivity and speed can be increased simultaneously.

### BOND’s seeds

For the design of BOND, we have employed multiple spaced seeds, as anticipated in the study of [34]. Properly designed, multiple spaced seeds can achieve near 100% sensitivity and for this reason we used them in BOND. However, the distribution of the matches in seeds is by no means random and finding the optimal arrangement is a hard problem [35]. A number of software programs for designing spaced seeds exist (most notably [36,37]) but SpEED [38,39] is the only polynomial-time algorithm that computes good (near optimal) seeds and we have adapted it for our needs. We have computed a w9s8 seed whose sensitivity is plotted in Figure 1(b) (BOND seed); its sensitivity is called “seed sensitivity” to avoid confusion with the sensitivity of the oligos). Its sensitivity at 75% identity is 98.3594% and the probability of detecting bad oligos of length 50 so that they respect condition (*C*_75_) is 99.8649%. (That is, the ratio between the area of the green region in Figure 1(b) and the total area of sequence identity 75% or higher; red and green together.) This is nearly perfect. We could have computed better seeds but this one did not make any error in all our tests and decreasing weight or increasing the number of seeds would slow down the program.

There are two reasons why this w9s8 seed is so effective. First, the sensitivity as given by the model is slightly underestimating the actual probability of the seed to find bad oligos since sequence identity below 75% is allowed in the model but not in our application. Second, we screen first the input data using a different w10s8 seed, also computed by SpEED, in order to quickly eliminate long repetitions.

### BOND’s algorithm

There are several features that make BOND fast. First, in order to achieve the sensitivities of the seeds as computed above, we need to investigate every possible hit in the input data, for each seed. In this context, a hit of a seed is a pair of substrings of the same length as the seed which match on all positions corresponding to 1’s in the seed. This would be time consuming for the w9s8 seed. Therefore, in Phase I, we filter the input data for long repeats, using the w10s8 seed. Moreover, only pairs of consecutive hitting substrings are considered.

Assume *L *is the required oligo length. A region of length *L *will be called *unique *if it does not have 75% sequence identity or more with any other region of the same length. We keep a binary array, initialized with 0’s, where starting positions of non-unique regions are eliminated by marking them with 1’s. Phase I usually eliminates a substantial portion of the input. Phase II uses the more sensitive w9s8 seed. We still do not consider all remaining 0’s as it would still be too time consuming. Instead, we use an effective heuristic. The middle position of the longest contiguous stretch of 0’s is considered first to be tested by the w9s8 seed. All possible hits concerning the potential oligo ending at this position are considered, thus using the entire power of the seed. If this oligo is found to be bad, then the position is marked with a 1 and the algorithm continues trying to find a good oligo until either one is found or the gene is shown not to have one. It is important to notice that the order in which the genes are considered is irrelevant; this helps parallelizing the process.

Using *T*_*m *_to denote the melting temperature, assume that the requested oligo length is *L* and the size of the melting temperature interval is *ΔT*_*m*_. For example, in our tests *L *= 50 and *ΔT*_*m *_= 10. By *L-mer *we shall mean a substring of length *L*. The high-level structure of the algorithm of BOND is as follows: 

1. Encode DNA sequences

2. Eliminate *L*-mers not satisfying (*C*_15_) 

3. Eliminate *L*-mers not satisfying GC content 

4. Eliminate *L*-mers not satisfying (*C*_75_) – Phase I 

5. Compute melting temperature interval *T *

6. Eliminate *L*-mers with *T*_*m*_ not in *T *

7. Check selected oligos for (*C*_75_) – Phase II 

8. Output oligos found, with genes and positions

*L*-mers are eliminated when found not to fulfill one of the required conditions. At any moment in the algorithm, the *L*-mers that were not eliminated so far are called *valid*. In step 1, the input data is processed so additional information is eliminated and only DNA sequences are kept. At the same time, all *L*-mers containing ambiguous or unknown nucleotides are eliminated. Step 2 eliminates *L*-mers containing 15-mers that occur elsewhere (condition (*C*_15_)). Step 3 eliminates *L*-mers with GC content outside the required range. Step 4 performs Phase I of (*C*_75_), using the w10s8 seed as explained above. For step 5, the melting temperatures (*T*_*m*_) are computed for all currently valid *L*-mers. The interval *T*, of size *ΔT*_*m*_, containing the highest number of genes having valid *L*-mers with *T*_*m*_ within *T *is computed by evaluating, in linear time, all intervals of the given size starting at any hundredth of degree between minimum and maximum *T*_*m*_. All *L*-mers having their melting temperature outside *T *are eliminated in step 6. Phase II of (*C*_75_) follows at step 7, using the full power of the w9s8 seed as explained above. Finally, at step 8, good oligos are reported.

### Evaluation algorithm

Evaluating the quality of oligos involves solving problems similar with those faced during oligo design and the lack of tools for the latter implied the nonexistence of adequate procedures for the former. For instance, the evaluation of [1] uses BLAST with default weight 11, thus having the problems we have mentioned above. We have designed a similar approach for our evaluation procedure, however, in order to accurately measure the performance of oligo design programs, including BOND, we need a much more powerful tool. We have used again SpEED to design the evaluation seed with the estimated probability of error 0.0001%. (This is computed as above, as the ratio between the red area and red plus green together. The areas are not shown for this seed, as the red one would be too small.) The sensitivity of the three seeds are compared in Figure 1(c). This seed is estimated to make one error for each million oligos but, again, it is even more accurate in practice. It is thus safe to assume that it provides perfect evaluation.

All hits of this seed in each oligo are checked against the entire input. Due to very low weight of the seed, the evaluation is time consuming, but the results are quite reliable. It is interesting to notice that our evaluation is still considerably faster than the exhaustive search of YODA. This is due to the performance of the multiple spaced seed and efficient implementation.

The evaluation seed accurately classifies the oligos into “good” or “bad”, depending on whether they satisfy conditions (*C*_75_) and (*C*_15_) or not, respectively. The ratio between bad oligos and the total number of oligos gives the specificity. In order to evaluate coverage, that is, how many of the maximum possible number of genes can be associated with good oligos, we give an algorithm that approximates very well this maximum number of genes. Due to, again, lack of tools, coverage has not been measured before.

The approximation of the maximum number of genes that can have oligos, needed to evaluate coverage, is done as follows. What we need to find is the melting temperature interval that can produce the highest number of oligos. Steps 1-4 of BOND are performed first. Then, for each interval *M *of size *ΔT*_*m*_, the number of genes that contain a valid *L*-mer with melting temperature in *M *is computed. Those intervals for which this number is less than the number of oligos computed by BOND are eliminated, since they have no chance to perform better than BOND. An interval larger than *ΔT*_*m *_is left and step 6 of BOND is performed with this interval. From the remaining oligos, an extended Phase II is performed, in which all valid *L*-mers are checked using the w9s8 seed. Finally, the highest number of genes in an interval of size *ΔT*_*m *_is reported.

### Implementation

Both BOND and the evaluation algorithms are given a fast parallel implementation. There are two level of parallelization, one involving bit operations and the other multiple processors.

DNA sequences are stored using two bits per nucleotide. All *L*-mers including unknown and ambiguous positions are eliminated from the beginning. Seeds are stored as 64-bit integers and hash tables are used to store the positions of all possible spaced *w*-mers corresponding to each seed; given a seed of weight *w*, a *spaced w-mer *is any sequence of *w *letters in positions that corresponds to the matches in the seed. Each spaced *w*-mer is computed by AND-ing the mask of the seed with each position in the DNA sequences and stored as a 64-bit integers. A similar strategy is used for 15-substrings (condition (*C*_15_)). Since only elimination is performed, there is no need to use a text index, such as the suffix array to sort the 15-mers.

The most time consuming tasks are then performed in parallel. The hash tables for all seeds are computed in parallel for all seeds in each set. The uniqueness checking for all candidate oligos is done in parallel as well.

## Results and discussion

### Datasets and parameters

The web site of PICKY [11] contains a wide variety of datasets and we considered them all for our comparison. We have added the dataset used in the survey of [1], consisting of 1421 genes involved in the development of the mouse nervous system, as well as the complete set of mouse genes from which those have been extracted. The datasets are shown in Table 1. Descriptions of the datasets, including download links are provided in the Additional file 1: Table S1.

**Table 1:** Datasets used for comparison

**Organism**	**Size**	**Genes**
Arabidopsis thaliana	36,298,530	28,952
Bee	6,010,949	11,324
C.elegans	34,753,016	30,935
Chicken	32,732,911	26,236
Drosophila melanogaster	32,198,758	18,962
E.coli	4,843,471	5,317
Human	72,720,516	28,205
Maize	38,963,590	58,579
Mouse	68,604,317	35,284
Plasmodium falciparum	10,739,506	9,518
Rice	113,204,455	66,710
Yeast	9,074,997	6,702
Zebrafish	23,003,650	12,238
Mouse RNA	93,830,285	36,598
Mouse 1421	4,354,947	1,421
TOTAL	581,333,898	376,981

The size given represents only the actual DNA sequences.

We have used the most common parameters for evaluating the programs: oligo length 50, GC content between 30..70%, size of melting temperature interval (*ΔT*_*m*_) 10, maximum identity with non-target below 75%, and maximum consecutive matches allowed 14.

### Comparison

**Figure 2:**
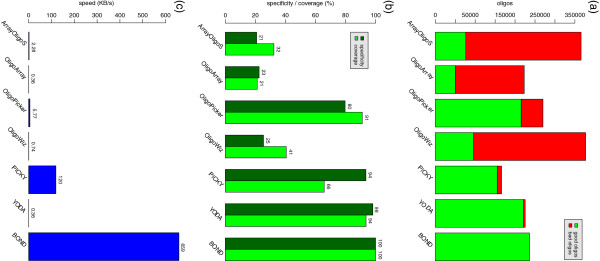
**Software programs comparison. **Comparison between BOND and the existing top six programs with respect to the quality of the oligos produced and speed. Plot **(a)** gives good versus bad oligos, **(b) **gives the specificity (ratio of good out of total oligos) and coverage (ratio of good out of the estimated maximum number of oligos), and **(c) **shows the speed in kilobytes of input data per second. In plots (b) and (c) the values are shown on top of the bars.

We have compared BOND with six programs that performed the best in the survey of [1]: ArrayOligoSelector [6], OligoArray [8], OligoPicker [10], OligoWiz [20], PICKY [11], and YODA [17]. Since the length of the oligos is fixed and the interval of melting temperatures has the same size, similar sensitivity is expected. Therefore, we have measured the specificity, coverage, and speed. Table 2 shows the total results for all seven programs. Detailed results, for each of the considered organisms, are given in the Additional file 2: Table S2. Visual comparison is presented in Figure 2, where (a) gives good vs bad oligos, (b) specificity and coverage, and (c) speed.

**Table 2:** Comparison of oligo design programs

**Program**	**Total oligos**	**Bad oligos**	**Good oligos**	**Specificity (%)**	**Coverage(%)**	**Time (s)**	**Speed (KB/s)**
ArrayOligoSelector	365,571	289,103	76,468	20.92	32.38	249,467	2.28
OligoArray	223,103	172,817	50,286	22.54	21.30	1,568,961	0.36
OligoPicker	269,971	54,715	215,256	79.73	91.16	98,343	5.77
OligoWiz	376,965	281,080	95,885	25.44	40.61	762,536	0.74
PICKY	166,126	10,697	155,429	93.56	65.83	4,745	119.64
YODA	225,332	4,190	221,142	98.14	93.66	1,577,385	0.36
BOND	236,111	0	236,111	100.00	100.00	862	658.59

The specificity represents the percentage of good oligos out of the total number. The maximum number of genes that have good oligos was estimated by our evaluation program at 236,122 oligos. The coverage represents the percentage the number of good oligos represents out of this estimated maximum.

All programs were tested on the same machine, Dell Vostro, with Intel Core i7-2600 CPU (3.40GHz, 4 cores, 8 threads), 16 GB of RAM, and 8MB of cache running GNU Linux version 2.6.38.8-desktop-69mib; the compiler used was gcc 4.4.3. The more time consuming evaluation was performed on the SHARCNET high performance computers (http://www.sharcnet.ca).

Out of the six existing programs, ArrayOligoSelector, OligoArray, and OligoWiz showed low performance in all respects. OligoPicker has fair coverage but the specificity and speed are both low. PICKY is the fastest and the specificity is good, however the coverage is low. PICKY uses the suffix array [40] to correctly fulfill condition (*C*_15_) and achieves good speed. YODA has the highest specificity and coverage, which is expected due to the exhaustive search involved. However, its speed is the lowest among all programs. For instance, it took more than five days to complete the rice dataset.

BOND performs significantly better than all the other programs; under the testing conditions, it achieves 100% sensitivity and 100% coverage. All 236,112 oligos produced are good and the estimated maximum possible is 236,123, that is, BOND may have missed 11 oligos. Likely, this is not due to lack of sensitivity but to the slightly suboptimal decision on the melting temperature interval (see Methods). As far as speed is concerned, BOND is six times faster than PICKY, two orders of magnitude faster than OligoPicker, and over three orders of magnitude faster than YODA. BOND completed most tests under one minute.

BOND is freely available for non-commercial use from http://www.csd.uwo.ca/\~ilie/BOND/. It requires no additional software and is easy to use. A wide range of input parameters allow the user to customize the oligos been produced in many ways: oligo length, GC content, size of melting temperature interval, oligos at 3’- or 5’-end, maximum number of consecutive matches allowed, maximum sequence identity allowed, secondary structure parameters. Details are given in the user manual provided in the same website.

## Conclusion

We provide an improved solution to the important problem of oligonucleotide design. Our BOND software provides highly specific oligos, that cover the highest possible number of genes, while running orders of magnitude faster than existing software. We have provided also a thorough evaluation of oligo design programs.

We have focused here on the basic problem of finding gene-specific oligos. We plan to extend BOND in the near future to compute genome-wide tiling arrays; see [1] for references to such programs.

We hope BOND will become a useful tool for researchers in biological and medical sciences by making the microarray procedures faster and more accurate. We shall gladly receive their suggestions for further development.

## Competing interests

The authors declare that they have no competing interests.

## Authors’ contributions

LI designed the new approach, the algorithm of BOND, the bit-parallel implementation, the evaluation algorithm, optimized the code, modified SpEED to compute the multiple spaced seeds used, and wrote the manuscript. HM wrote the parallel code of BOND and the evaluation program, helped improving its running speed, and performed the tests and comparisons with the other programs. GBG was the advisor on biological matters and WFS provided algorithmic suggestions. All authors met regularly to discuss decisions concerning the development of the software. The final version of the manuscripts was read and approved by all authors.

## Supplementary Material

Additional file 1: Table S1 Descriptions of the datasets used in the paper, including download links. Click here for file

Additional file 2: Table S2 Detailed results for comparison between BOND and the currently leading software programs for oligo design. Click here for file
