# Supplementary material for: BOND: Basic OligoNucleotide Design
Source: BMC Bioinformatics. 2013 Feb 27;14:69. doi: 10.1186/1471-2105-14-69 (PMC3648450; doi:10.1186/1471-2105-14-69)
Supplement: Additional file 1: Table S1 — Descriptions of the datasets used in the paper, including download links. [file 1471-2105-14-69-S1.pdf]

Supplementary Table 1: Descriptions of the data sets used in the paper, including download links. Most datasets that were used in our tests can also be downloaded from the website of PICKY and all from the website of BOND:
`www.complex.iastate.edu/download/Picky/Picky2_oligos/data/`
`www.csd.uwo.ca/~ilie/BOND/`

**Organism**	**Dataset description**<br>**Link**
Arabidopsis thaliana	complete CDS sequences from TIGR<br>ftp://ftp.tigr.org/pub/data/a_thaliana/ath1/SEQUENCES/
Bee	EST sequences,TIGR<br>ftp://ftp.ncbi.nih.gov/genomes/Apis_mellifera/
C. elegans	EST sequences, TIGR<br>ftp://ftp.ncbi.nih.gov/genomes/Caenorhabditis_elegans/
Chicken	mRNA sequences, UCSC Genome Bioinformatics<br>http://hgdownload.cse.ucsc.edu/goldenPath/galGal2/bigZips/
Drosophila melanogaster	complete CDS sequences, Flybase<br>ftp://flybase.net/genomes/Drosophila_melanogaster/current/fasta/
E. coli	gene sequences, NCBI<br>ftp://ftp.ncbi.nih.gov/genomes/Bacteria/Escherichia_coli_O157H7_EDL933/
Human	mRNAs sequences, NCBI human repository<br>ftp://ftp.ncbi.nih.gov/genomes/H_sapiens/RNA/
Maize	EST sequences, TIGR<br>ftp://ftp.tigr.org/pub/data/z_mays/
Mouse	cDNA sequences, Ensembl<br>ftp://ftp.ensembl.org/pub/current_mouse/data/fasta/cdna/
Plasmodium falciparum	gene sequences, TIGR (release 8)<br>ftp://ftp.tigr.org/pub/data/tgi/Plasmodium_falciparum
Rice	cDNA genes, TIGR<br>ftp://ftp.tigr.org/pub/data/Eukaryotic_Projects/o_sativa/annotation_dbs/pseudomolecules<br>/version_5.0/all.chrs/
Yeast	CDS sequences, Saccharomyces Genome Database<br>ftp://genome-ftp.stanford.edu/pub/yeast/data_download/sequence/genomic_sequence/orf_dna/
Zebrafish	mRNA sequences, UCSC Genome Bioinformatics.<br>http://hgdownload.cse.ucsc.edu/goldenPath/danRer2/bigZips/
Mouse RNA	complete gene set including Mouse 1421, NCBI<br>ftp://ftp.ncbi.nih.gov/refseq/M_musculus/mRNA_Prot/
Mouse 1421	1421 mouse genes used in the survey of Lemoine *et al.*<br>pers. comm. to L.I. from Stephane Le Crom
