# Supplementary material for: BOND: Basic OligoNucleotide Design
Source: BMC Bioinformatics. 2013 Feb 27;14:69. doi: 10.1186/1471-2105-14-69 (PMC3648450; doi:10.1186/1471-2105-14-69)
Supplement: Additional file 2: Table S2 — Detailed results for comparison between BOND and the currently leading software programs for oligo design. [file 1471-2105-14-69-S2.pdf]

**Supplementary Table 2: Detailed results for comparison between BOND and the currently leading software programs for oligo design**

| DATASETS | | | Maximum | ArrayOligoSelector | | | | | | OligoArray | | | | | | OligoPicker | | | | | |
|---|---|---|---|---|---|---|---|---|---|---|---|---|---|---|---|---|---|---|---|---|---|
| Organism | Size | Genes | oligos* | Total | Bad | Good | Spec. (%) | Cov. (%) | Time (s) | Total | Bad | Good | Spec. (%) | Cov. (%) | Time (s) | Total | Bad | Good | Spec. (%) | Cov. (%) | Time (s) |
| **Arabidopsis** | **36,298,530** | **28,952** | **21,332** | 27,918 | 22,641 | 5,277 | 18.90 | 24.74 | 11,017 | 19,340 | 16,771 | 2,569 | 13.28 | 12.04 | 53,030 | 23,687 | 6,107 | 17,580 | 74.22 | 82.41 | 8,387 |
| **Bee** | **6,010,949** | **11,324** | **10,675** | 11,317 | 1,359 | 9,958 | 87.99 | 93.28 | 3,132 | 10,575 | 4,136 | 6,439 | 60.89 | 60.32 | 3,662 | 10,823 | 354 | 10,469 | 96.73 | 98.07 | 594 |
| **C.elegans** | **34,753,016** | **30,935** | **21,724** | 30,788 | 25,057 | 5,731 | 18.61 | 26.38 | 14,350 | 23,142 | 19,503 | 3,639 | 15.72 | 16.75 | 76,475 | 24,086 | 4,116 | 19,970 | 82.91 | 91.93 | 5,027 |
| **Chickem** | **32,732,911** | **26,236** | **16,692** | 26,036 | 20,357 | 5,679 | 21.81 | 34.02 | 10,222 | 15,984 | 12,136 | 3,848 | 24.07 | 23.05 | 62,202 | 17,485 | 1,366 | 16,119 | 92.19 | 96.57 | 2,995 |
| **Drosophila** | **32,198,758** | **18,962** | **11,826** | 16,501 | 10,590 | 5,911 | 35.82 | 49.98 | 8,022 | 10,034 | 5,803 | 4,231 | 42.17 | 35.78 | 52,325 | 12,245 | 802 | 11,443 | 93.45 | 96.76 | 2,343 |
| **E.coli** | **4,843,471** | **5,317** | **4,647** | 5,161 | 647 | 4,514 | 87.46 | 97.14 | 1,213 | 4,503 | 1,124 | 3,379 | 75.04 | 72.71 | 2,569 | 4,672 | 145 | 4,527 | 96.90 | 97.42 | 260 |
| **Human** | **72,720,516** | **28,205** | **18,781** | 27,923 | 26,288 | 1,635 | 5.86 | 8.71 | 22,114 | 18,083 | 16,589 | 1,494 | 8.26 | 7.95 | 87,132 | 21,410 | 4,056 | 17,354 | 81.06 | 92.40 | 8,307 |
| **Maize** | **38,963,590** | **58,579** | **43,614** | 58,522 | 38,224 | 20,298 | 34.68 | 46.54 | 23,629 | 42,423 | 30,736 | 11,687 | 27.55 | 26.80 | 60,612 | 49,475 | 9,835 | 39,640 | 80.12 | 90.89 | 16,123 |
| **Mouse** | **68,604,317** | **35,284** | **20,200** | 34,491 | 31,848 | 2,643 | 7.66 | 13.08 | 27,869 | 20,353 | 18,164 | 2,189 | 10.76 | 10.84 | 310,259 | 23,779 | 5,275 | 18,504 | 77.82 | 91.60 | 9,997 |
| **Plasmodium** | **10,739,506** | **9,518** | **4,527** | 8,991 | 7,455 | 1,536 | 17.08 | 33.93 | 4,490 | 5,370 | 4,225 | 1,145 | 21.32 | 25.29 | 49,186 | 5,964 | 1,671 | 4,293 | 71.98 | 94.83 | 925 |
| **Rice** | **113,204,455** | **66,710** | **28,556** | 66,520 | 62,797 | 3,723 | 5.60 | 13.04 | 76,180 | 19,814 | 17,857 | 1,957 | 9.88 | 6.85 | 398,915 | 38,692 | 14,756 | 23,936 | 61.86 | 83.82 | 31,106 |
| **Yeast** | **9,074,997** | **6,702** | **6,178** | 6,645 | 1,922 | 4,723 | 71.08 | 76.45 | 2,423 | 6,156 | 3,016 | 3,140 | 51.01 | 50.83 | 5,160 | 6,249 | 208 | 6,041 | 96.67 | 97.78 | 540 |
| **Zebrafish** | **23,003,650** | **12,238** | **7,989** | 8,481 | 6,215 | 2,266 | 26.72 | 28.36 | 6,764 | 7,573 | 5,233 | 2,340 | 30.90 | 29.29 | 33,309 | 8,226 | 579 | 7,647 | 92.96 | 95.72 | 1,478 |
| **Mouse RNA** | **93,830,285** | **36,598** | **17,963** | 34,856 | 33,529 | 1,327 | 3.81 | 7.39 | 37,266 | 18,343 | 17,209 | 1,134 | 6.18 | 6.31 | 373,770 | 21,757 | 5,428 | 16,329 | 75.05 | 90.90 | 10,195 |
| **Mouse 1421** | **4,354,947** | **1,421** | **1,418** | 1,421 | 174 | 1,247 | 87.76 | 87.94 | 776 | 1,410 | 315 | 1,095 | 77.66 | 77.22 | 355 | 1,421 | 17 | 1,404 | 98.80 | 99.01 | 66 |
| **TOTAL** | **581,333,898** | **376,981** | **236,122** | **365,571** | **289,103** | **76,468** | **20.92** | **32.38** | **249,467** | **223,103** | **172,817** | **50,286** | **22.54** | **21.30** | **1,568,961** | **269,971** | **54,715** | **215,256** | **79.73** | **91.16** | **98,343** |

| DATASETS | | | Maximum | OligoWiz | | | | | | PICKY | | | | | | YODA | | | | | |
|---|---|---|---|---|---|---|---|---|---|---|---|---|---|---|---|---|---|---|---|---|---|
| Organism | Size | Genes | oligos* | Total | Bad | Good | Spec. (%) | Cov. (%) | Time (s) | Total | Bad | Good | Spec. (%) | Cov. (%) | Time (s) | Total | Bad | Good | Spec. (%) | Cov. (%) | Time (s) |
| **Arabidopsis** | **36,298,530** | **28,952** | **21,332** | 28,952 | 16,635 | 12,317 | 42.54 | 57.74 | 19,048 | 18,584 | 3,447 | 15,137 | 81.45 | 70.96 | 436 | 20,318 | 271 | 20,047 | 98.67 | 93.98 | 291,012 |
| **Bee** | **6,010,949** | **11,324** | **10,675** | 11,324 | 1,544 | 9,780 | 86.37 | 91.62 | 6,396 | 10,442 | 110 | 10,332 | 98.95 | 96.79 | 62 | 10,526 | 136 | 10,390 | 98.71 | 97.33 | 4,133 |
| **C.elegans** | **34,753,016** | **30,935** | **21,724** | 30,935 | 18,617 | 12,318 | 39.82 | 56.70 | 20,572 | 16,807 | 1,012 | 15,795 | 93.98 | 72.71 | 393 | 20,941 | 406 | 20,535 | 98.06 | 94.53 | 91,543 |
| **Chickem** | **32,732,911** | **26,236** | **16,692** | 26,235 | 15,466 | 10,769 | 41.05 | 64.52 | 20,073 | 13,781 | 315 | 13,466 | 97.71 | 80.67 | 422 | 16,306 | 236 | 16,070 | 98.55 | 96.27 | 44,447 |
| **Drosophila** | **32,198,758** | **18,962** | **11,826** | 18,962 | 10,701 | 8,261 | 43.57 | 69.85 | 22,780 | 10,484 | 304 | 10,180 | 97.10 | 86.08 | 493 | 11,613 | 163 | 11,450 | 98.60 | 96.82 | 37,967 |
| **E.coli** | **4,843,471** | **5,317** | **4,647** | 5,317 | 2,309 | 3,008 | 56.57 | 64.73 | 1,949 | 4,557 | 65 | 4,492 | 98.57 | 96.66 | 47 | 4,590 | 16 | 4,574 | 99.65 | 98.43 | 2,034 |
| **Human** | **72,720,516** | **28,205** | **18,781** | 28,205 | 25,119 | 3,086 | 10.94 | 16.43 | 86,908 | 10,807 | 495 | 10,312 | 95.42 | 54.91 | 392 | 17,997 | 163 | 17,834 | 99.09 | 94.96 | 122,281 |
| **Maize** | **38,963,590** | **58,579** | **43,614** | 58,579 | 48,243 | 10,336 | 17.64 | 23.70 | 30,986 | 26,506 | 1,757 | 24,749 | 93.37 | 56.75 | 442 | 41,198 | 1,285 | 39,913 | 96.88 | 91.51 | 205,416 |
| **Mouse** | **68,604,317** | **35,284** | **20,200** | 35,283 | 29,799 | 5,484 | 15.54 | 27.15 | 104,003 | 12,473 | 631 | 11,842 | 94.94 | 58.62 | 457 | 19,399 | 410 | 18,989 | 97.89 | 94.00 | 135,605 |
| **Plasmodium** | **10,739,506** | **9,518** | **4,527** | 9,517 | 7,811 | 1,706 | 17.93 | 37.69 | 41,828 | 4,138 | 131 | 4,007 | 96.83 | 88.51 | 43 | 3,885 | 55 | 3,830 | 98.58 | 84.60 | 3,745 |
| **Rice** | **113,204,455** | **66,710** | **28,556** | 66,710 | 60,551 | 6,159 | 9.23 | 21.57 | 249,976 | 13,365 | 1,468 | 11,897 | 89.02 | 41.66 | 617 | 25,891 | 704 | 25,187 | 97.28 | 88.20 | 457,827 |
| **Yeast** | **9,074,997** | **6,702** | **6,178** | 6,702 | 875 | 5,827 | 86.94 | 94.32 | 3,661 | 5,850 | 138 | 5,712 | 97.64 | 92.46 | 121 | 6,128 | 34 | 6,094 | 99.45 | 98.64 | 9,023 |
| **Zebrafish** | **23,003,650** | **12,238** | **7,989** | 12,238 | 8,810 | 3,428 | 28.01 | 42.91 | 47,075 | 7,428 | 382 | 7,046 | 94.86 | 88.20 | 345 | 7,855 | 76 | 7,779 | 99.03 | 97.37 | 29,956 |
| **Mouse RNA** | **93,830,285** | **36,598** | **17,963** | 36,585 | 34,456 | 2,129 | 5.82 | 11.85 | 103,987 | 9,483 | 406 | 9,077 | 95.72 | 50.53 | 445 | 17,267 | 235 | 17,032 | 98.64 | 94.82 | 141,190 |
| **Mouse 1421** | **4,354,947** | **1,421** | **1,418** | 1,421 | 144 | 1,277 | 89.87 | 90.06 | 3,294 | 1,421 | 36 | 1,385 | 97.47 | 97.67 | 30 | 1,418 | - | 1,418 | 100.00 | 100.00 | 1,206 |
| **TOTAL** | **581,333,898** | **376,981** | **236,122** | **376,965** | **281,080** | **95,885** | **25.44** | **40.61** | **762,536** | **166,126** | **10,697** | **155,429** | **93.56** | **65.83** | **4,745** | **225,332** | **4,190** | **221,142** | **98.14** | **93.66** | **1,577,385** |

| DATASETS | | | Maximum | BOND | | | | | |
|---|---|---|---|---|---|---|---|---|---|
| Organism | Size | Genes | oligos* | Total | Bad | Good | Spec. (%) | Cov. (%) | Time (s) |
| **Arabidopsis** | **36,298,530** | **28,952** | **21,332** | 21,325 | 0 | 21,325 | 100.00 | 99.97 | 45 |
| **Bee** | **6,010,949** | **11,324** | **10,675** | 10,675 | 0 | 10,675 | 100.00 | 100.00 | 6 |
| **C.elegans** | **34,753,016** | **30,935** | **21,724** | 21,724 | 0 | 21,724 | 100.00 | 100.00 | 44 |
| **Chickem** | **32,732,911** | **26,236** | **16,692** | 16,692 | 0 | 16,692 | 100.00 | 100.00 | 38 |
| **Drosophila** | **32,198,758** | **18,962** | **11,826** | 11,826 | 0 | 11,826 | 100.00 | 100.00 | 34 |
| **E.coli** | **4,843,471** | **5,317** | **4,647** | 4,647 | 0 | 4,647 | 100.00 | 100.00 | 4 |
| **Human** | **72,720,516** | **28,205** | **18,781** | 18,781 | 0 | 18,781 | 100.00 | 100.00 | 109 |
| **Maize** | **38,963,590** | **58,579** | **43,614** | 43,614 | 0 | 43,614 | 100.00 | 100.00 | 59 |
| **Mouse** | **68,604,317** | **35,284** | **20,200** | 20,200 | 0 | 20,200 | 100.00 | 100.00 | 99 |
| **Plasmodium** | **10,739,506** | **9,518** | **4,527** | 4,527 | 0 | 4,527 | 100.00 | 100.00 | 14 |
| **Rice** | **113,204,455** | **66,710** | **28,556** | 28,552 | 0 | 28,552 | 100.00 | 99.99 | 215 |
| **Yeast** | **9,074,997** | **6,702** | **6,178** | 6,178 | 0 | 6,178 | 100.00 | 100.00 | 9 |
| **Zebrafish** | **23,003,650** | **12,238** | **7,989** | 7,989 | 0 | 7,989 | 100.00 | 100.00 | 31 |
| **Mouse RNA** | **93,830,285** | **36,598** | **17,963** | 17,963 | 0 | 17,963 | 100.00 | 100.00 | 151 |
| **Mouse 1421** | **4,354,947** | **1,421** | **1,418** | 1,418 | 0 | 1,418 | 100.00 | 100.00 | 4 |
| **TOTAL** | **581,333,898** | **376,981** | **236,122** | **236,111** | **0** | **236,111** | **100.00** | **100.00** | **862** |

*Maximum oligos means the maximum number of genes that can have oligos.
